# Quantifying future Olympic sport selection: a data-driven framework for SDE evaluation and selection

**DOI:** 10.3389/fspor.2025.1596196

**Published:** 2025-07-29

**Authors:** Yunkun Song, Rui Dai, Qiaoyi Zhang, Yizhuo Sun

**Affiliations:** ^1^ Sendelta International Academy, Shenzhen, Guangdong, China; ^2^ School of Media, Yangtze University, Jingzhou, Hubei, China; ^3^ Department of Earth and Space Sciences, Southern University of Science and Technology, Shenzhen, Guangdong, China

**Keywords:** the olympic games and SDEs, scoring and labelling system, analytic hierarchy process, principal component analysis, *k*-nearest neighbour classifier

## Abstract

The Olympic Games are the world’s foremost sporting event, with over 200 countries participating. As the Games evolve, sports, disciplines, and events (SDEs) are periodically added or removed. The selection process for Olympic sports is inherently subjective, as seen with breakdancing’s inclusion in the 2024 Paris Olympics and exclusion from the 2028 Los Angeles Olympics. Thus, developing a quantitative decision-making model is crucial for the International Olympic Committee (IOC). This study evaluates IOC criteria for new sports by considering factors such as social media engagement, TV viewership across demographics, affordability, gender equity, youth appeal, cultural diversity, and global involvement. Our model employs a scoring and labelling system based on the Analytic Hierarchy Process (AHP), which calculates the relative importance of each factor. Using Principal Component Analysis (PCA) for feature extraction, we apply a *k*-nearest neighbour (KNN) classifier for further evaluation. We apply this model to assess potential SDEs for the 2032 Brisbane Olympics, considering their popularity in Australia and alignment with Olympic criteria. Our findings suggest that Esports, Australian rules football, and pickleball are the top candidates for inclusion, while tug of war, bowling, and chess are also recommended based on their historical relevance and global popularity.

## 1 Introduction

### 1.1 Background

The Olympic Games are the world’s largest and most prestigious sporting celebration, uniting over 200 countries to compete in a diverse array of events (1). Over more than a century, the Olympic program has undergone significant transformation. Traditionally, sports, disciplines, and events (SDEs) such as the marathon, gymnastics, and swimming have been the cornerstone of the Games (1, 2). These long-standing events embody the core Olympic values of excellence, friendship, and respect. Yet, as global society evolves, the need for the Olympic program to remain relevant and dynamic has become increasingly apparent.

In recent years, the International Olympic Committee (IOC) has actively sought to modernize the Games by adapting its program to the interests of a younger and more diverse audience (3). This strategic shift is exemplified by the Tokyo 2020 Olympics, where sports like karate, sport climbing, surfing, and skateboarding were introduced for the first time, signalling an effort to engage contemporary audiences and to reflect modern cultural trends (4). Moreover, the debut of breakdancing as an Olympic sport in Paris 2024 further underscores the IOC’s commitment to embracing unconventional and urban sports that resonate with today’s youth (5). Meanwhile, traditional events continue to be reviewed and adjusted, ensuring that the overall program remains vibrant, competitive, and reflective of current global interests.

Despite these progressive changes, the process of including or excluding sports remains complex and often subjective. The decision-making process is not only influenced by the sport’s global appeal but also by its relevance to the host country. For instance, the inclusion of baseball and softball at the Tokyo 2020 Olympics was partly driven by Japan’s deep cultural connection to these sports (6), whereas in previous editions, the removal of events such as wrestling or baseball/softball has sparked debates over fairness and transparency in the selection process (6). This subjectivity poses a significant challenge for the IOC as it strives to balance tradition with innovation in a rapidly evolving global sports landscape.

The growing complexity of the global sports ecosystem—with its myriad SDEs vying for recognition—has heightened the need for a more quantitative and systematic approach to evaluating potential Olympic events. It is imperative for the IOC to assess each proposed sport based on objective and measurable criteria rather than relying solely on subjective opinions or the preferences of individual stakeholders. A transparent and data-driven decision-making model would not only streamline the evaluation process but also bolster the legitimacy of the selection decisions.

Under the core policies of the IOC, factors such as global popularity, gender parity, youth engagement, and stringent anti-doping measures are paramount (7, 8). Popularity, which can be measured through metrics such as television viewership, social media engagement, and overall public interest, is a critical factor (9). Similarly, gender parity ensures that both male and female athletes are provided equal opportunities, reinforcing the ideals of inclusivity and fairness (8, 10). Youth engagement is crucial for sustaining the long-term appeal of the Games, as evidenced by the strategic inclusion of sports that attract younger audiences, like skateboarding and surfing (11). Additionally, robust anti-doping policies are essential to maintain the integrity and fairness of competition (12).

To address the inherent subjectivity and complexity of current decision-making processes, a more rational approach that leverages quantitative methods and data-driven insights is necessary. Developing a comprehensive scoring system—integrated within a decision-making model that accounts for these core criteria—would enable more objective assessments of potential new sports. Such a framework promises to reduce bias, provide clarity in the evaluation process, and ensure that the Olympic Games continue to serve as a fair and inclusive platform for athletes worldwide.

In this study, we present a scoring and classification framework that combines expert-informed weighting with data-driven techniques. Specifically, we employ Principal Component Analysis (PCA) and *k*-Nearest Neighbour (KNN) algorithms to support decision-making. PCA is a mathematical technique that simplifies complex datasets by identifying the most important patterns and reducing the number of variables, while preserving the essence of the data. In our case, it allows us to condense multiple evaluation criteria into a few key dimensions that reveal how different sports compare. KNN, on the other hand, is a straightforward method for classification. It operates on the principle that similar sports—those with comparable features—tend to belong to the same category. To determine whether a new sport aligns with current Olympic trends, KNN looks at the “nearest” existing sports and assigns a label based on the majority of their classifications. Together, these methods help minimize subjectivity in sport evaluation, enhance model transparency, and offer a replicable, data-supported approach to Olympic program planning.

A transparent and data-driven model is essential for streamlining the evaluation of potential Olympic sports and enhancing the credibility of selection decisions. However, beyond technical criteria, Olympic programme planning must also align with the broader mission of the Olympic Movement. As emphasized in the Olympic Charter and recent IOC initiatives like the Hamburg Declaration (13), the Games serve not only as a stage for elite competition but also as a global platform to promote values such as excellence, friendship, and respect (14), while addressing pressing global issues like physical inactivity and environmental sustainability (15). These principles underscore the need to balance sporting performance with long-term societal benefits in sport selection.

### 1.2 Problem restatement

The essential question is to develop a model to quantitatively evaluate each SDE according to the established criteria, providing further recommendations for the future Olympic programme. The question can be broken down into the following subquestions:

• Problem 1: We need to determine the key factors that need to be considered when evaluating SDEs based on the IOC’s criteria. These factors may be quantitative or qualitative. It is also necessary for us to collect relevant data of identified factors.
• Problem 2: Using the identified factors in Problem 1, we need to construct a mathematical model to evaluate SDEs within the scope of the Olympic criteria. The proposed model should be applicable to evaluate different SDEs and can return the most suitable ones that align well with the criteria.
• Problem 3: We need to test our model with various SDEs, focusing especially on those which were added or removed from recent Olympics or that have continuously been in the Olympic program since the 1988 Games or earlier. In addition, we should also highlight how the model applies to these diverse SDEs and discuss how it supports or refutes their current Olympic status.
• Problem 4: We aim to identify three additional SDEs for the 2032 Olympics based on their scores. Furthermore, it is also interesting to provide recommendations for new SDEs that can be considered for inclusion in the Olympics for 2036 or beyond as well.

## 2 Assumptions and notations

### 2.1 Assumptions and justifications

To help determine the model scope, in this paper we adopt several assumptions listed as below:

• Assumption 1: For each SDE, we mainly focus on the representative leagues or the most famous events and players. Because the largest events or most popular athletes typically have the greatest impact, complete data statistics and provide the most significant insights for modelling.
• Assumption 2: The location of where the SDE is held is not a major impact on the decision-making process. Decision-making of SDEs is typically accomplished and centralized by the IOC. Besides, SDEs held in different regions often share similar features in terms of the Olympic criteria, while athletes and audiences are provided with similar facilities and accommodations.
• Assumption 3: The basic rules of SDEs remain unchanged in a certain period of time. While it is true that some SDEs may introduce trial innovations, the basic rules of SDEs are highly unlikely to change significantly. Since athletes are trained based on stable rules, and such consistency ensures that audiences will remain engaged.
• Assumption 4: The SDEs should provide equal chance for men and women to participate. Following the gender-equal principles and the Olympic Agenda 2020+5 (16), it is reasonable to assume that all the SDEs should prioritize gender equity and ensure equal representation of men and women.

### 2.2 Symbols and notations

In this paper, we mainly use lowercase letters a for scalars, boldface letters a for vectors, and uppercase letters A for matrices. More details of symbols and notations used in our paper are listed in Table 1.

**Table 1:** Symbols and notations used in the paper and explanations.

Notation	Explanation
ASMF	Average social media followers of top five players or athletes (m)
EL	Expense level
MR	Male ratio
FR	Female ratio
T5MAI	Top5 male athletes average income (m)
T5FAI	Top5 female athletes average income (m)
WHIL	Whether have international league
CP	Countries play
APOP	Age proportion of players
ALCR	Safety level considering risk of injury, training requirements and protection
FPL	Fair play (level)
SL	Sustainability (level)
D	Dataset of original SDEs with feature vectors and labels
$D_{\;p}$	Dataset of extracted features after PCA transformation
N	Number of samples in the dataset
d	Dimension of feature vector
k	Number of nearest neighbours
x	Constructed feature vector of length d
X	Constructed feature matrix of size d×N, each column represents a feature vector
$X_{ij}$	Element in the i-th row and j-th column of the feature matrix
$\hat{X}$	Feature matrix after data rescaling and normalization
${\hat{X}}_{\mathrm{centered}}$	Feature matrix after data centralization
C	Covariance matrix
$U_{p}$	PCA projection matrix consisting of top p eigenvectors
${\hat{X}}_{p}$	Matrix representing the linear transformation after PCA projection
$x_{q}$	Query feature vector of test SDE data
$\mathrm{dist}(x_{i},s_{j})$	The Euclidean distance between two feature vectors $x_{i}$ and $x_{j}$

## 3 Methodology

### 3.1 Model overview

**Figure 1:**
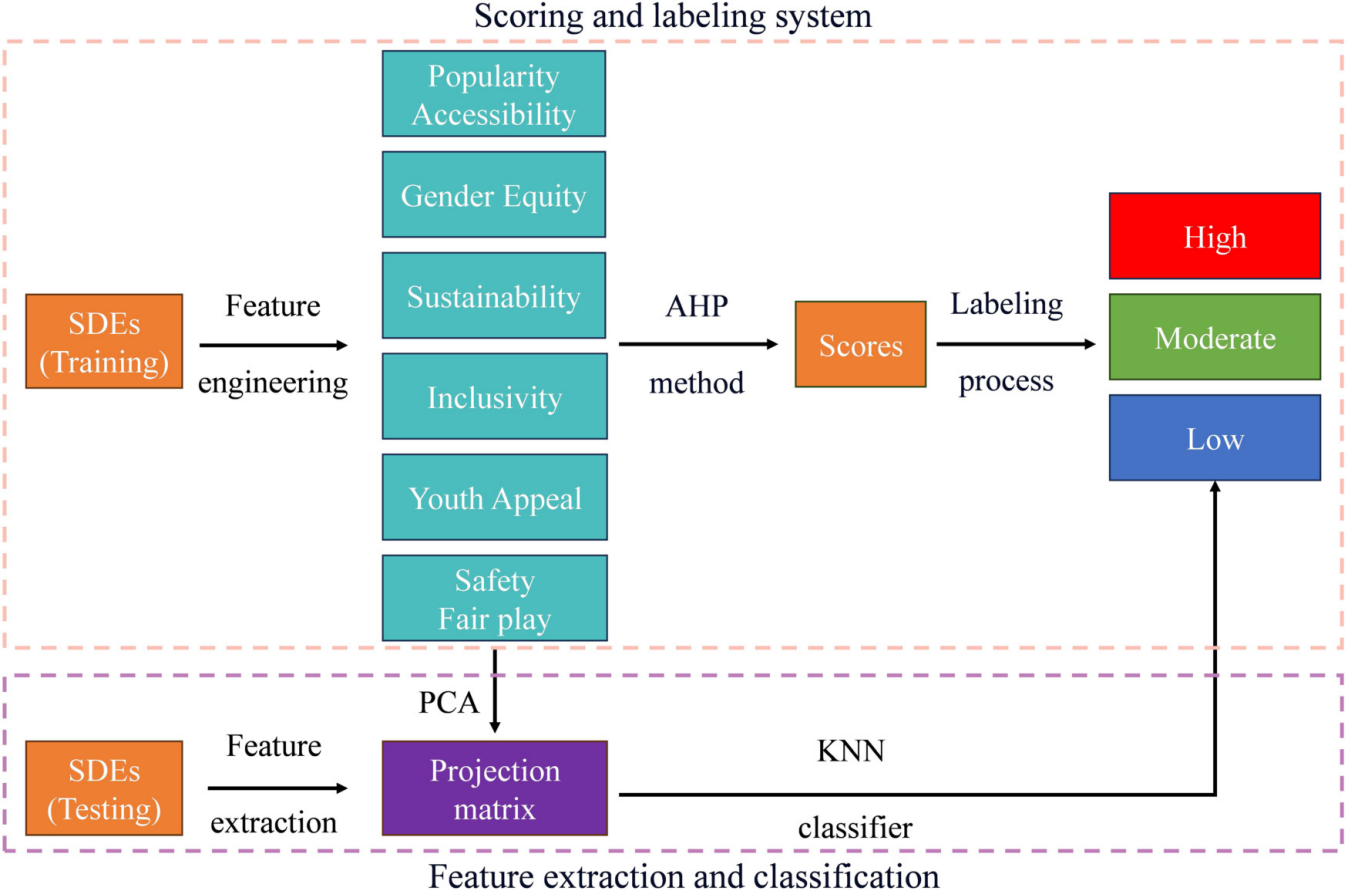
Flowchart of the proposed scoring-based classification model.

In this paper, to address the concerns of the IOC, we propose a scoring-based classification model, which consists of two main building blocks, namely “the scoring and labelling system,” and the “feature extraction and classification phase.” The flowchart of our model is illustrated in Figure 1.

### 3.2 Scoring and labelling

To assign scores to different SDEs,we develop a scoring and labelling system, including four consecutive steps: identification of important factors, feature engineering, AHP method and labelling process.

#### 3.2.1 Identification of important factors: question 1

In this section, we investigate and identify important factors related to the IOC criteria of new SDE inclusion for the Olympic Games. Our data is collected based on publicly available database, reports and research papers (17–20).

• Popularity and accessibility. To measure popularity, we use the average number of top five athletes’ social media followers, which is a quantitative and deterministic variable and the unit is million. For the accessibility, we consider the affordability level in terms of costs of equipment and new constructions qualitative variable ranging from 1 to 5. The larger the value, the higher the accessibility. For example, benefiting from a huge fan base and relatively low cost, football, basketball and table tennis are considered highly popular and accessible. While the average number of social media followers among top athletes provides a proxy for global visibility and youth engagement, it may not fully represent grassroots or amateur-level popularity. Actual participation metrics, such as the number of registered athletes or nationwide participation rates, could serve as valuable complements, but are not uniformly available across sports. This limitation is noted, and future research may incorporate broader participation indicators when data access improves.
• Gender equity. The indicators for gender equality in SDEs lies in two different aspects. First, we use the ratio of professional female players to male players in major leagues/events to determine the participation factor, which is a quantitative and deterministic variable. Besides, since gender inequality is also evident in terms of the income gap, it is measured by the income ratio of the top five male and female athletes.
• Sustainability. Sports events and activities may inevitably produce carbon emissions and resource waste. Besides,the new construction of sports facilities is also associated with significant environmental impact. Therefore, we can use qualitative variable to rate sustainability level from 1 to 5. The sustainability of a SDE is considered better with higher ratings.
• Inclusivity. To evaluate inclusivity of a given SDE, we consider two factors: the number of countries that frequently host or broadcast the competition, and if it has famous leagues or events in at least 4 continents. We can use qualitative variable to rate these two factors. We recognize that measuring inclusivity solely through the international presence of broadcasting and hosting may bias toward media-oriented sports. Therefore, while this approach captures global visibility and infrastructure readiness, it does not fully account for community-level engagement. The inclusion of metrics such as the number of participating countries in international federations or athlete registration data would provide a more comprehensive view and should be considered in future model updates.
• Relevance and innovation. As youth appeal and engagement is playing a more and more important role in the Olympic Games, thus we investigate and analyze the TV audiences, which are divided into different age groups. We use the ratio of audiences under 35 years as an indicator for this criteria, which is a quantitative and deterministic variable.
• Safety and fair play. For the safety level, we consider the risk of injury, training requirements and protection measurements. For the fair play level, we collect historical data of the doping records. These two factors are evaluated using qualitative variables ranging from 1 to 5.

Table 2 lists statistics of 6 represented sports according to different criteria and identified factors. From Table 2, we make several interesting observations, as follows,

• Basketball has the highest popularity and accessibility in terms of a large audience base and relatively low cost compared to more expensive sports such as sailing and golf, in that basketball is played in almost every country with famous international leagues and organizations like FIBA and NBA. Besides, basketball has simple equipment requirements, making it affordable for many ordinary people.
• Gymnastics enjoys the highest level of gender equity as it includes distinct sets of apparatuses and events tailored to female players, which increases their media attention and income.
• Golf has the lowest sustainability score among all sports, since it has high water consumption, occupies vast amounts of land, which may eventually lead to deforestation or destruction of natural habitats. Besides, as the maintenance and construction of golf courses will generate high carbon emissions, it is also considered energy-intensive.
• Golf, sailing and shooting have lower youth appeal because the pace of the game is slow, and the cost of specialized equipment can be very high.

**Table 2:** Statistics of identified factors of representative sports.

Criteria	Factor	Basketball	Shooting	Sailing	Gymnastics	Tennis	Golf
Popularity and accessibility	Social media followers (m)	120	1	1	20	100	15
	Affordability (level)	4	2	1	3	1	1
Gender equity	Participant ratio (f/m)	0.053	0.429	0.251	1.50	0.668	0.251
	Income ratio (f/m)	0.152	0.500	0.668	2.06	0.471	0.268
Sustainability	Environmental impact (level)	3	4	4	3	3	1
Inclusivity	Number of countries	200	100	100	150	150	120
	Famous leagues in at least 4 continents (Y/N)	1	1	0	1	1	1
Relevance and innovation	Youth appeal	0.60	0.30	0.25	0.55	0.6	0.25
Safety and fair play	Safety (level)	3	5	3	2	4	5
	Fair play (level)	3	5	5	3	3	5

The summarization and statistics of relevant factors can help us better understand the development and operations of different SDEs.

#### 3.2.2 AHP method: question 2

**Figure 2:**
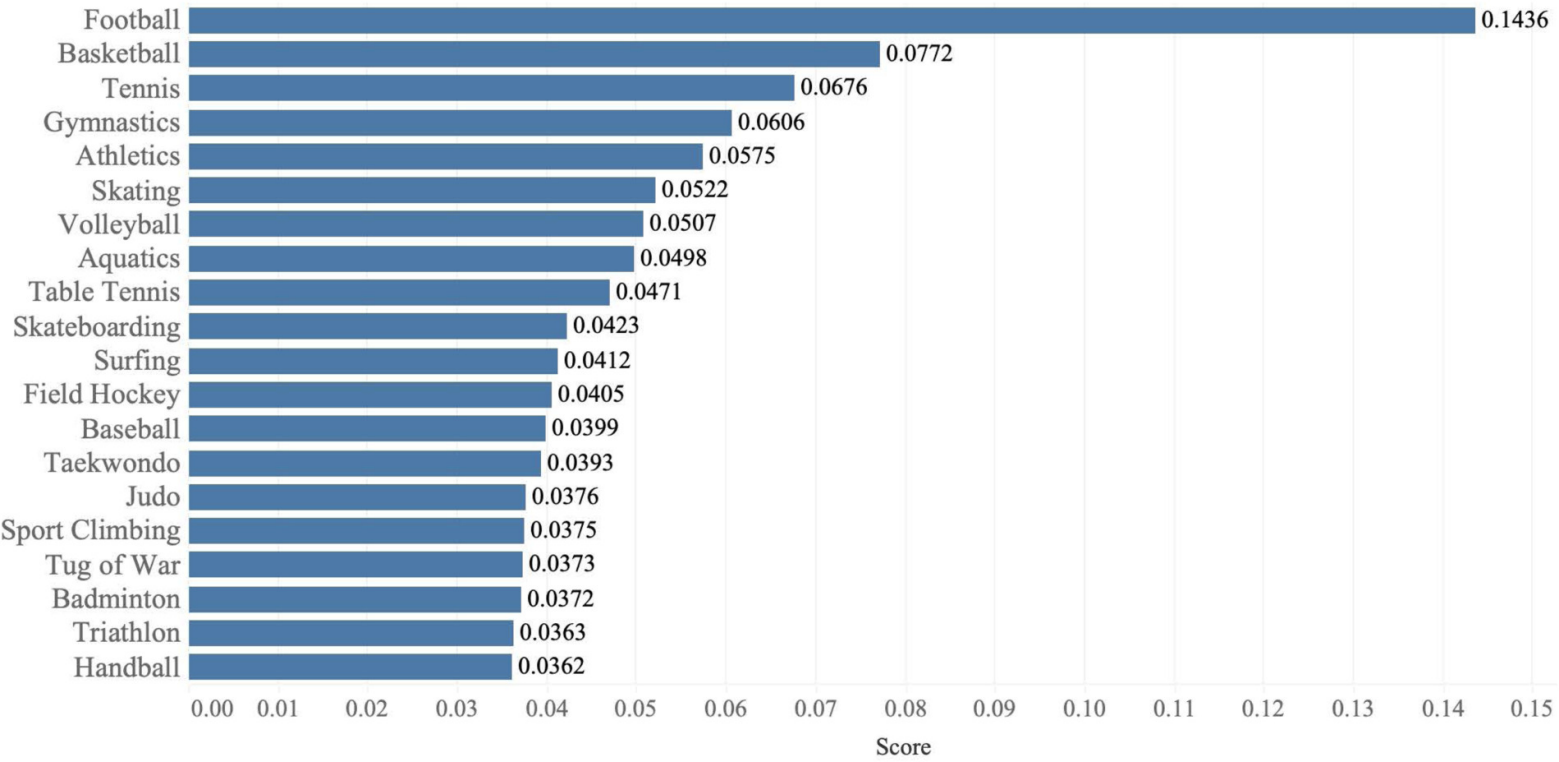
Results of top 20 SDEs based on our scoring scheme.

To address the concerns of IOC for SDE evaluations, we propose to adopt the AHP method (21) and complete our scoring system. Based on our analysis, we identify top 5 SDEs which align best with the IOC criteria: football (soccer), basketball, gymnastics, tennis and volleyball, by using AHP method. Figure 2 shows the calculated scores of top 20 SDEs, and more details are given in this section.

**Figure 3:**
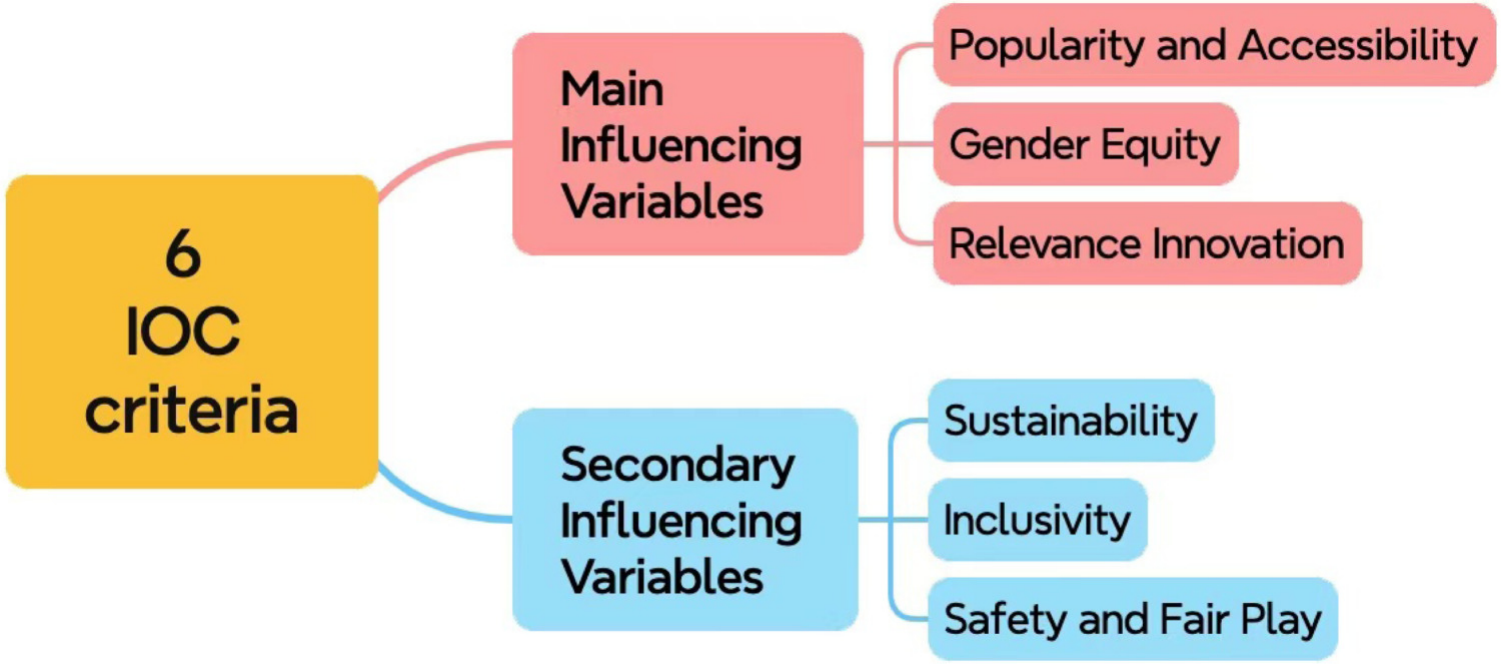
List of IOC criteria.

**Defining the criteria.** Based on the Olympic Agenda 2020 (16), the goal of IOC is to prioritize youth engagement, gender-balance, and innovation. Hence as illustrated in Figure 3, we can divide the six decisive criteria into two categories, namely the major and minor criteria. Briefly, the main criteria includes popularity and accessibility, gender equity and relevance and innovation, while the sub-criteria consists of sustainability, inclusivity and safety and fair play.

More specifically, according to Andrew Moore, “The Olympics are unlike any other sporting event in the world because of their capacity to unite people through a shared enthusiasm for sport on a global scale,” the importance of popularity and accessibility is thus underlined. Besides, the Paris 2024 sets a milestone as the first Olympic Games to achieve full gender parity (22), which indicates the significance of gender equity for SDEs. In addition, as the Olympic Games tend to introduce more SDEs related to young people, such as the breaking, BMX freestyle, skateboarding and 3×3 basketball events, innovations and youth appeal also plays an important role. For sub-criteria, safety and fair play should be regarded as the one with the highest proportion among three of them, since Olympian’s mindset is to exhibit integrity and positive character in all aspects of sport and in life. Therefore, the weight of size relationship between these six criteria are popularity and accessibility ≻ gender equity ≻ relevance ≻ safety and fair play ≻ sustainability ≻ inclusivity.

**Figure 4:**
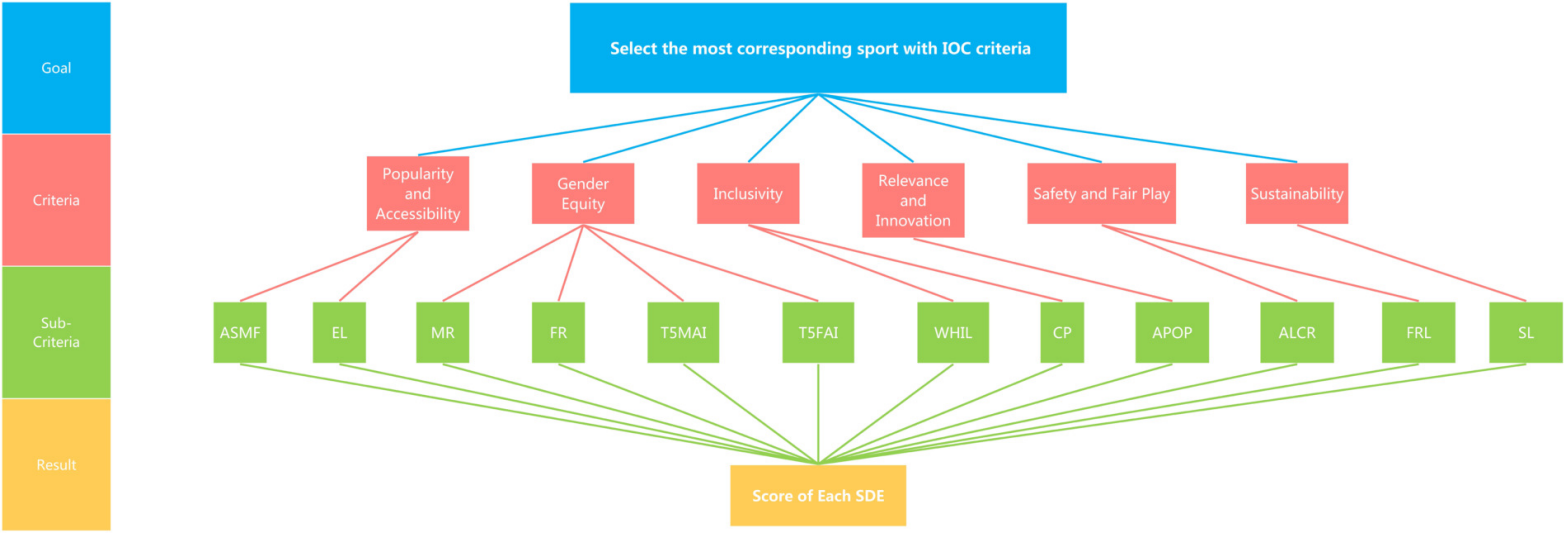
Illustration of different criteria and hierarchy structure.

**Figure 5:**
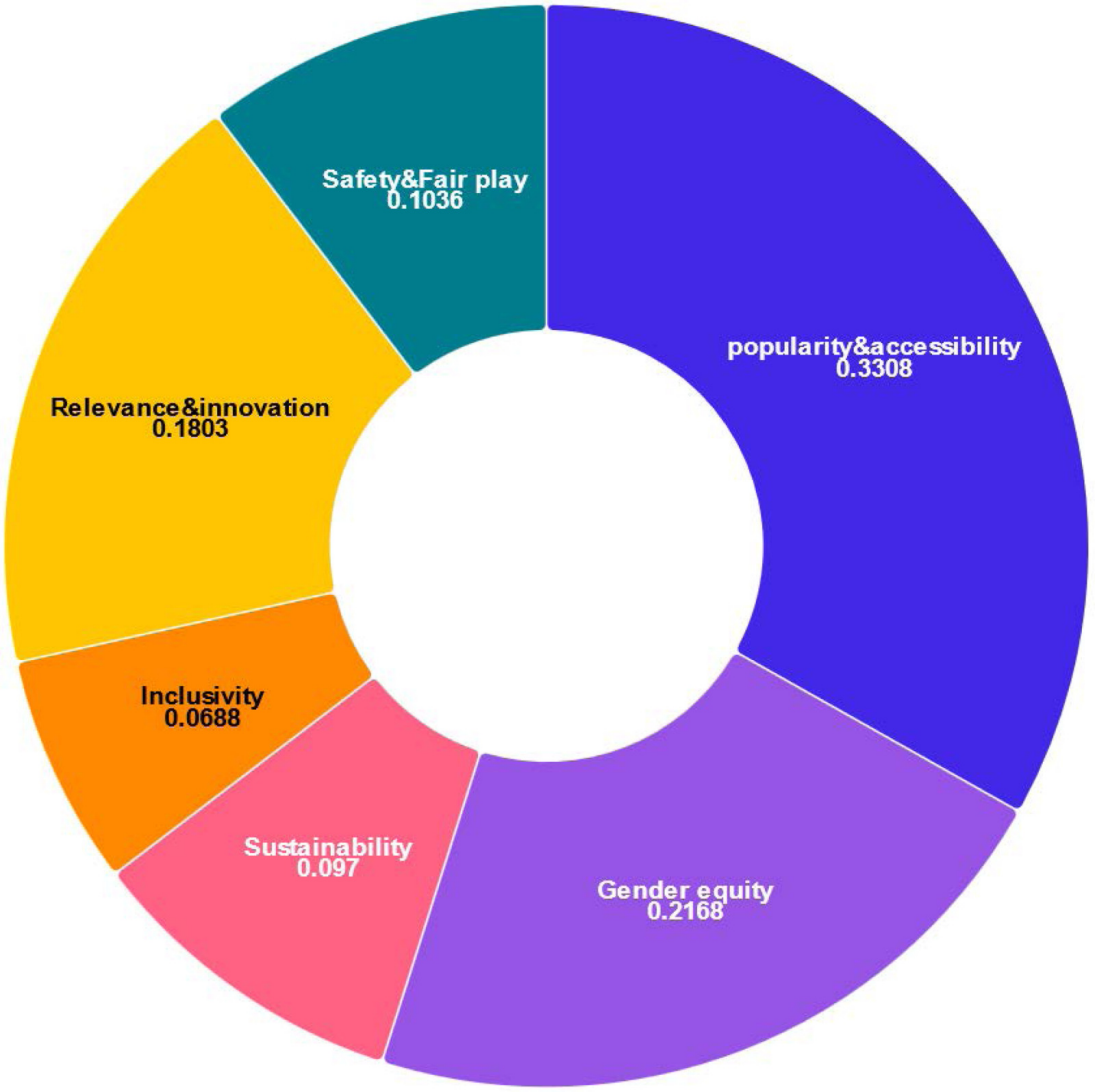
Illustration of weights on different criteria.

• Hierarchy structure. The AHP method allows us to assess the relative weight of multiple criteria against given criteria in an intuitive manner. A hierarchy structure of variables is illustrated in Figure 4.
• Pairwise comparison matrix. To perform pair-wise comparison, we construct a 6 by 6 comparison matrix A to characterize relative preference in each compared pair using a 1–5 scale for relative importance. For example, if popularity and accessibility is regarded more significant than inclusivity, then the popularity and accessibility-inclusivity value will be 4, which indicates that popularity and accessibility is considered 4 times as important as inclusivity. Table 3 presents details of the constructed comparison matrix A.
• Priority vector. After establishing the comparison matrix A, we then calculate the priority vector w, which represents the relative weights of the criteria. Mathematically, w can be obtained via
  (1) $$Aw=\lambda_{\max}w$$
  where $\lambda_{\max}$ is the largest eigenvalue of A. Then, w is further normalized so that the sum of its elements equals 1. The results of weights on are illustrated in Figure 5 following Equation 1.
• Consistency check. Once weights are obtained, it is necessary to check the consistency. Inevitably, the final matrix of criteria may be subject to inconsistency to varying degrees, because the numerical values are derived from the subjective preferences. Therefore, to ensure the consistency we compute the consistency index (CI) via
  (2) $$CI=\frac{\lambda_{\max}-n}{n-1},$$
  where n refers to the number of criteria. Based on CI from Equation 2 and Random Index (RI), the consistency ratio (CR) can be calculated via
  (3) $$CR=\frac{CI}{RI}.$$
  If CR<0.1, then the comparisons are considered consistent. The first 16 random consistency index is listed in Table 4. By applying SPSSAU (23), we confirm that our CR value calculated based on Equation 3 is much lower than 0.1, thereby demonstrating the consistency of the constructed comparison matrix A.

**Table 3:** Constructed comparison matrix based on pair-wise relationship among different variables.

Criteria	Popularity and accessibility	Gender equity	Sustainability	Inclusivity	Relevance and innovation	Safety and fair play
Popularity and accessibility	1.00	2.00	3.00	4.00	3.00	2.00
Gender equity	0.50	1.00	3.00	4.00	1.00	2.00
Sustainability	0.33	0.33	1.00	2.00	0.50	1.00
Inclusivity	0.25	0.25	0.50	1.00	0.33	1.00
Relevance and innovation	0.33	1.00	2.00	3.00	1.00	2.00
Safety and fair play	0.50	0.50	1.00	1.00	0.50	1.00

**Table 4:** Random consistency index.

n	3	4	5	6	7	8	9	10	11	12	13	14	15	16
RI	0.52	0.89	1.12	1.26	1.36	1.41	1.46	1.49	1.52	1.54	1.56	1.58	1.59	1.5943

#### 3.2.3 Feature engineering

**Figure 6:**
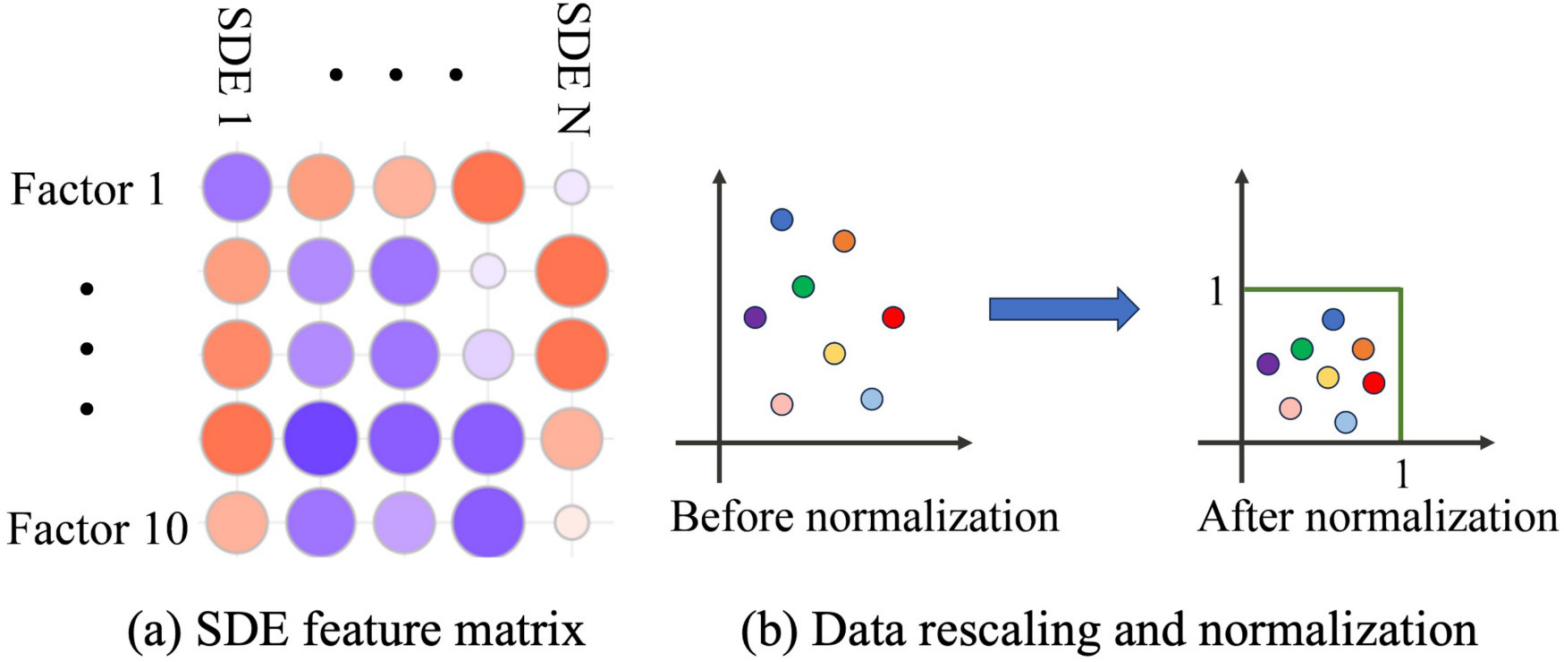
Illustration of weights on different criteria. **(a)** SDE feature matrix and **(b)** Data rescaling and normalization.

Although the scoring mechanism may be useful to rate certain SDEs, it can be affected by subjectivity and noise in data collection and weight decision. As can be seen from Figure 2, we notice that SDEs of similar scores may share common features. Therefore, based on the 10 identified factors of IOC criteria listed in previous sections and Table 2, we can construct feature vectors for SDEs. Each SDE can be described as a feature vector x of length 10, then all feature vectors are stacked into a large feature matrix $\hat{X}=[x_{1},x_{2},\ldots ,x_{N}]\in R^{10\times N}$, as presented in Table 2 and Figure 6a. From Table 2, the factors are measured on different scales, thus to get rid of the scale and let the model focus on patterns in data, we apply row-wise normalization via

(4) $$\hat{X}(i,\;:\;)=\frac{X(i,\;:\;)}{\sum_{\;j=1}^{N}X_{ij}},\;\forall i=1,2,\ldots ,10$$

where X(i,:) and $\hat{X}(i,\;:\;)$ represents the i-th row of the original and the normalized data, respectively. Equation 4 rescales each factor such that the sum of its resulting elements is 1, which convert raw frequencies or values into discrete probability distributions. The data rescaling and normalization process is illustrated in Figure 6b.

#### 3.2.4 Labelling

After obtaining the weighted scores of SDEs, we can create labels accordingly. Briefly, we rank the scores in descending order and classify SDEs in three different categories of priority: High, Moderate and Low. The corresponding labels for High, Moderate and Low ratings are 1, 0, and −1, respectively. Therefore, the SDE dataset can be represented by $D=\{(x_{1},y_{1}),(x_{2},y_{2}),\ldots ,(x_{N},y_{N})\}$, where $y_{i}\in \{1,0,-1\}$ is the corresponding label.

• High. The top 12 SDEs are labelled “High,” which reflects their widespread global appeal across different age groups, significant media coverage and importance in the Olympics. Sports such as swimming, gymnastics, and basketball fall in this category.
• Moderate. SDEs ranked between 13 and 27 are labelled “Moderate,” which describes SDEs that are popular but may not have as global reach or as large a fan base compared to the top SDEs. The Moderate SDEs include Judo, handball and Archery.
• Low. The rest are categorized as “low,” due to their limited international participation, gender inequality or doping concerns. For example, flag football and fencing have a more regional following compared to basketball. Besides, as weightlifting suffers from doping and corruption scandals, it is also rated low by our scoring system.

The scoring and labelling system provides us with training/testing samples and corresponding labels that can be used to learn patterns and relationships in the data. Therefore, we convert the original problem as a classification task with three different classes or categories: High, Moderate and Low.

### 3.3 Feature extraction and classification

#### 3.3.1 Feature extraction via principal component analysis

Given the scores of SDEs, a straightforward way to determine the category of a new SDE is to directly compare its score with the existing SDEs. However such naive comparison can be easily affected by change of data, variations of criteria and weights. To reduce subjectivity, we utilize the principal component analysis (PCA) (24, 25) to capture the most important features. Specifically, we start by centering the normalized data $\hat{X}$ via

(5) $${\hat{X}}_{\mathrm{centered}}=\hat{X}-\mu$$

where μ is the mean vector calculated as

(6) $$\mu =\sum_{i=1}^{N}{\hat{x}}_{i}$$

Then, according to Equations 5 and 6, we can obtain the covariance matrix C that characterizes pair-wise relationships via

(7) $$C=\frac{1}{N}{\hat{X}}_{\mathrm{centered}}*{\hat{X}}_{\mathrm{centered}}^{T}$$

Following the results of Equation 7, we can obtain the eigenvectors U and eigenvalues λ of C by applying PCA. After sorting the eigenvalues in descending order, we then select the top p eigenvectors as the projection matrix $U_{\;p}\in R^{10\times p}$ via

(8) $$U_{p}=[u_{1},u_{2},\ldots ,u_{p}]$$

The low-dimensional feature embeddings ${\hat{X}}_{\;p}\in R^{\;p\times N}$ is obtained by projecting the normalized data $\hat{X}$ onto the selected principal components $U_{\;p}$ from Equation 8 via

(9) $${\hat{X}}_{\;p}=U_{\;p}^{T}\hat{X}$$

It is noticed that PCA is an unsupervised feature extraction method, thus when new SDEs are considered, they can also be included to update the projection matrix in an incremental manner to improve the quality of feature learning and dimensionality reduction.

#### 3.3.2 K-nearest neighbour classifier

**Figure 7:**
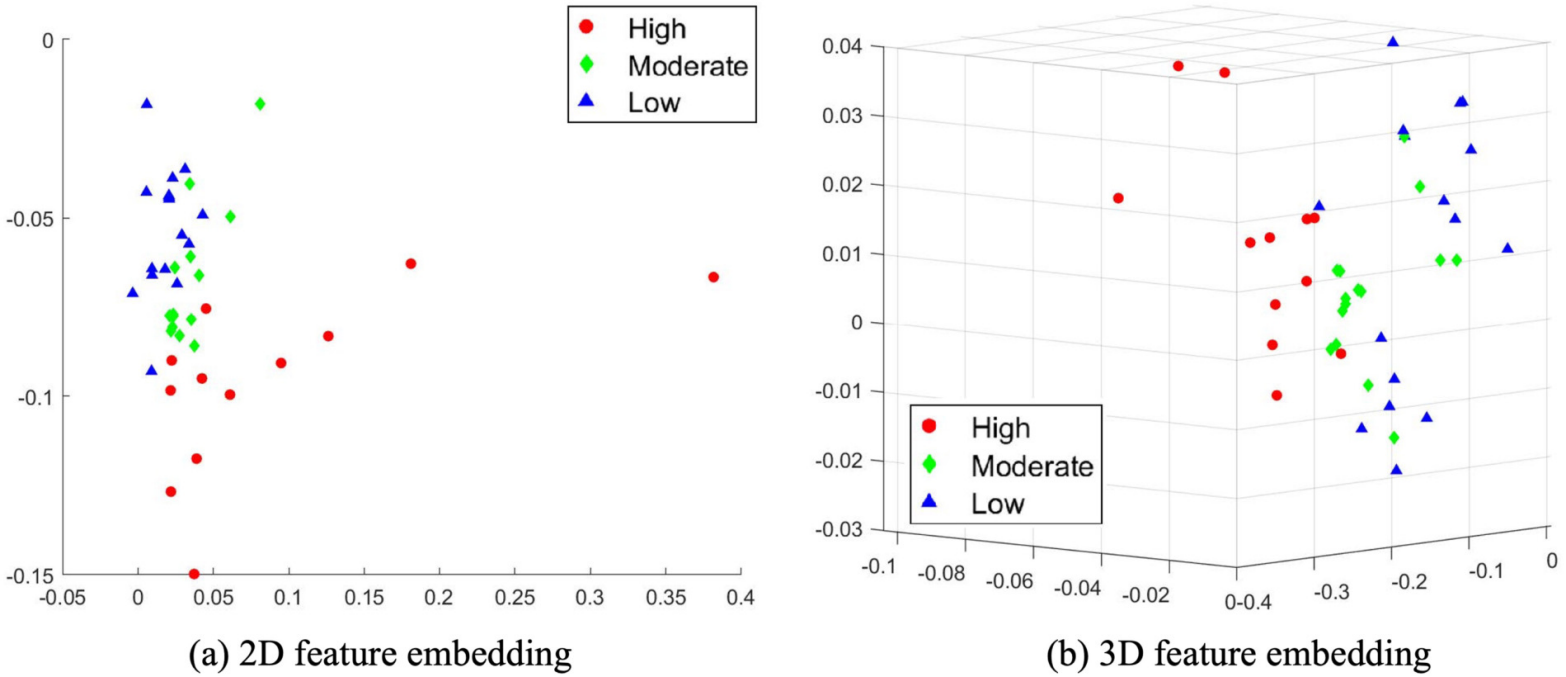
Visualization of PCA feature embeddings of SDEs. **(a)** 2D feature embedding and **(b)** 3D feature embedding.

Based on the features ${\hat{X}}_{p}$ extracted by PCA, the two-dimensional and three-dimensional feature embeddings of SDEs are visualized in Figure 7. It can be seen that SDEs falling from high and low categories are separated, while the majority of moderate samples are close to each other. Furthermore, the decision boundary between classes is highly irregular and non-linear, thus in addition to the SDE scores, we also take advantage of the K-nearest neighbour (KNN) classifier (26) to determine the class label of test SDEs.

Specifically, given the PCA-based training data $D_{\;p}=\{({\hat{x}}_{\;p_{1}},y_{1}),({\hat{x}}_{\;p_{2}},y_{2}),\ldots ,({\hat{x}}_{\;p_{N}},y_{N})\}$ and a query SDE feature vector $x_{q}\in R^{10}$, we normalize the query vector and obtain its PCA projection ${\hat{x}}_{q}\in R^{\;p}$ based on Equations 4, 9, respectively. Then we calculate the similarity between ${\hat{x}}_{q}$ and each data point ${\hat{x}}_{\;pi}$ using the Euclidean distance metric d via

(10) $$\mathrm{dist}({\hat{x}}_{q},{\hat{x}}_{\;p_{i}})=\|{\hat{x}}_{q}-{\hat{x}}_{\;p_{i}}{\|}_{2}$$

Then, following Equation 10, we are able to extract from $D_{p}$ the k-nearest neighbours of ${\hat{x}}_{q}$, which are denoted by $N_{k}=\{({\hat{x}}_{q_{1}},y_{q_{1}}),({\hat{x}}_{q_{2}},y_{q_{2}}),\ldots ,({\hat{x}}_{q_{k}},y_{q_{k}})\}$. Based on the identified k neighbours, we can assign a label $y_{q}$ to ${\hat{x}}_{q}$ by performing majority voting that returns the class with the most votes.

#### 3.3.3 Complexity analysis

The feature extraction and classification method is briefed in Algorithm 1. The computational burden of the proposed method lies mainly in two parts, namely the PCA feature extraction and the KNN classifier. The computational complexity of PCA is $O(Np^{2}+p^{3})$, which consists of deriving covariance matrix and eigenvectors. The computational complexity of KNN is O(Np), which involves calculating Euclidean distances for all N samples. Therefore, the total computational complexity is $O(Np^{2}+p^{3})$ for the proposed method. Since the matrix multiplication and KNN neighbour search can both be parallelized, the algorithm may be more efficient by adopting parallel computing techniques.

**Algorithm 1:** The PCA-based KNN classifier.

• Input: SDE dataset $D_{p}$, PCA matrix $U_{p}$, query SDE feature vector ${\hat{x}}_{q}$, number of neighbours k. • Output: Estimated label $y_{q}$. • Step 1 (PCA projection): Calculate the extracted feature ${\hat{x}}_{q}$ via ${\hat{x}}_{q}=U_{\;p}^{T}x_{q}$. • Step 2 (KNN search): Calculate the Euclidean distance between ${\hat{x}}_{q}$ and all samples of $D_{p}$ according to Equation 9, and then identify its k nearest neighbours $N_{k}$. • Step 3 (Majority voting): The predicted class label $y_{q}$ is determined by the voting for the nearest neighbour $N_{k}$ and the majority class label is assigned $y_{q}$.

## 4 Experiments

In this section, we mainly report results of our experiments, which are performed with MATLAB2024a on a moderate computer equipped with Core(TM) i5 @ 2.9 GHz and 16 GB RAM.

### 4.1 Experimental settings

Dataset: We collected a comprehensive dataset D consisting of N=50 samples covering a wide range of different SDEs, such as basketball, skating, fencing and cycling.

Parameters: There are several key parameters of the proposed PCA-based KNN classification model. Specifically, P decides the low-dimensional embeddings of feature vectors and k determines the number of nearest neighbours of the KNN classifier. In our experiments, p is chosen based on 95% explained variance, and k is chosen from 3 to 8 using grid search.

### 4.2 Model testing: question 3

#### 4.2.1 Evaluations of recently added or removed Olympic SDEs

To evaluate SDEs that have been added or removed from recent Olympics, we consider 4 different SDEs as our test set $D_{\mathrm{test}}$, including Breakdancing, cricket, flag football and basketball (3×3), and the est are used for training. Briefly, breakdancing was introduced in the 2024 Paris Olympics but will be excluded in the 2028 Los Angeles Olympics. Cricket will be added to the 2028 Olympics since its first appearance in 1900. Similarly, flag football will also be included, which will become its first debut in the Olympics. Besides, basketball (3×3) was introduced in the 2020 Tokyo Olympics and will also be included in the next Olympics.

**Figure 8:**
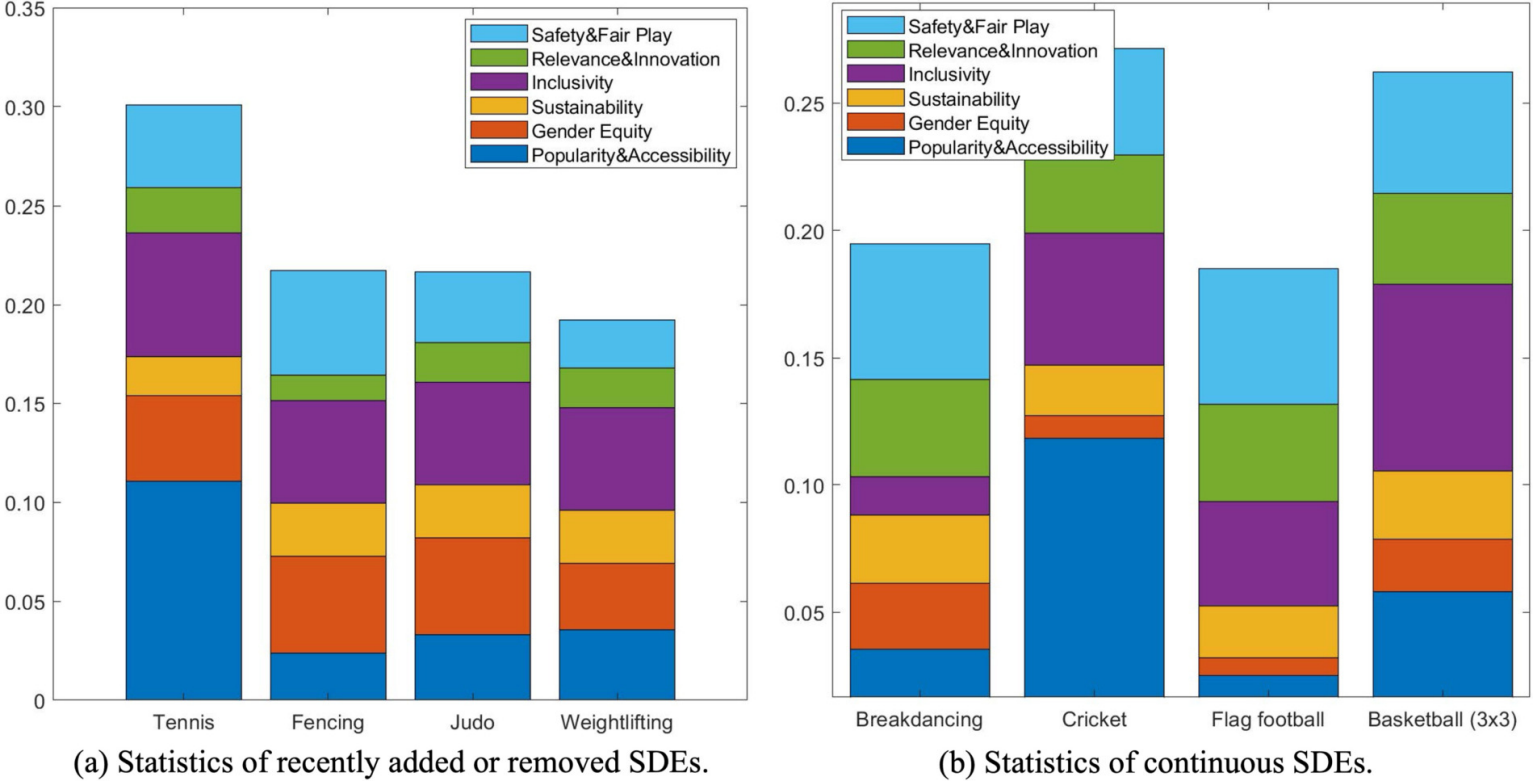
Statistics of **(a)** recently added or removed SDEs and **(b)** continuous SDEs.

First, we utilize our scoring system to derive the feature vectors and corresponding scores of the selected 4 SDEs. Then we can apply our PCA-based KNN model to obtain the estimated labels. The results are shown in Figure 8a and Supplementary Table S1. It can be seen that our scoring system and proposed classification method can effectively and accurately characterize the current status of selected SDEs. Specifically, Breakdancing and flag football are labelled “Low” according to our model’s predictions. Interestingly, breakdancing will be removed and flag football is not currently included. Besides, Cricket and Basketball are labelled “High” since they have a large audience base and enjoy high level of inclusivity. It is noticed that Basketball (3×3) was added in 2020 Tokyo Olympics and Cricket will return to the Olympics in 2028. Our results are inconsistent with both the development of the SDEs and their current status in modern Olympics.

#### 4.2.2 Evaluations of continuous Olympic SDEs

To evaluate SDEs that have continuously been in the Olympic programme, we also choose 4 representative sports as our test set: Tennis, Fencing, Judo and Weightlifting. These sports were selected to represent a diverse range of characteristics relevant to the IOC evaluation framework, including differences in gender equity, global inclusivity, youth appeal, and fair play issues. In addition, they have each been continuously included in the Olympic programme for a substantial period, having been introduced or reintroduced since 1988, 1896, 1972, and 1920, respectively. The selected SDEs all have a relatively long history but they also face different challenges. Similarly, the rest of SDEs are used for training the PCA projection matrix and KNN classifier. Following the same feature extraction and classification steps, we report the results in Figure 8b and Supplementary Table S2.

As shown in Figure 8b, Tennis is assigned a much higher score compared to other sports, due to its large audience base, tremendous market value, high level of gender equity and also better projection measurements. According to our system and model, fencing is considered more suitable that Judo for the Olympic programme because it has a higher level of inclusivity and accessibility. Besides, its fast-paced competition also appeals to younger audience. Interestingly, although weightlifting is considered one of the longest continuous sports, it is assigned a “Low” label by our system and the proposed model. According to Figure 8b, we notice that the low score of weightlifting results from low gender equity and poor fair play level due to increasing doping concerns (27).

### 4.3 Future Olympic SDEs: question 4

As the development of our society, the future Olympics need to adapt to changing global dynamics, audience expectations, and technology advancements. Therefore, for the 2032 Brisbane Olympic Games, we investigate 6 different new SDEs as strong candidates: netball, Australian rules football, Esports, darts, snooker and pickleball. The first two sports—netball and Australian rules football—were selected based on their strong cultural significance and widespread popularity within Australia, the host nation. To capture a balance between local cultural relevance and international appeal, the remaining four sports were chosen for their alignment with broader global trends: Esports has experienced explosive global growth, particularly among younger audiences, and has established professional leagues worldwide. Darts and snooker maintain large international fan bases, long-standing professional circuits, and strong media appeal. Pickleball, though relatively new, has seen rapid growth in participation across multiple continents and is recognized for its inclusivity and accessibility, especially among diverse age groups.

**Figure 9:**
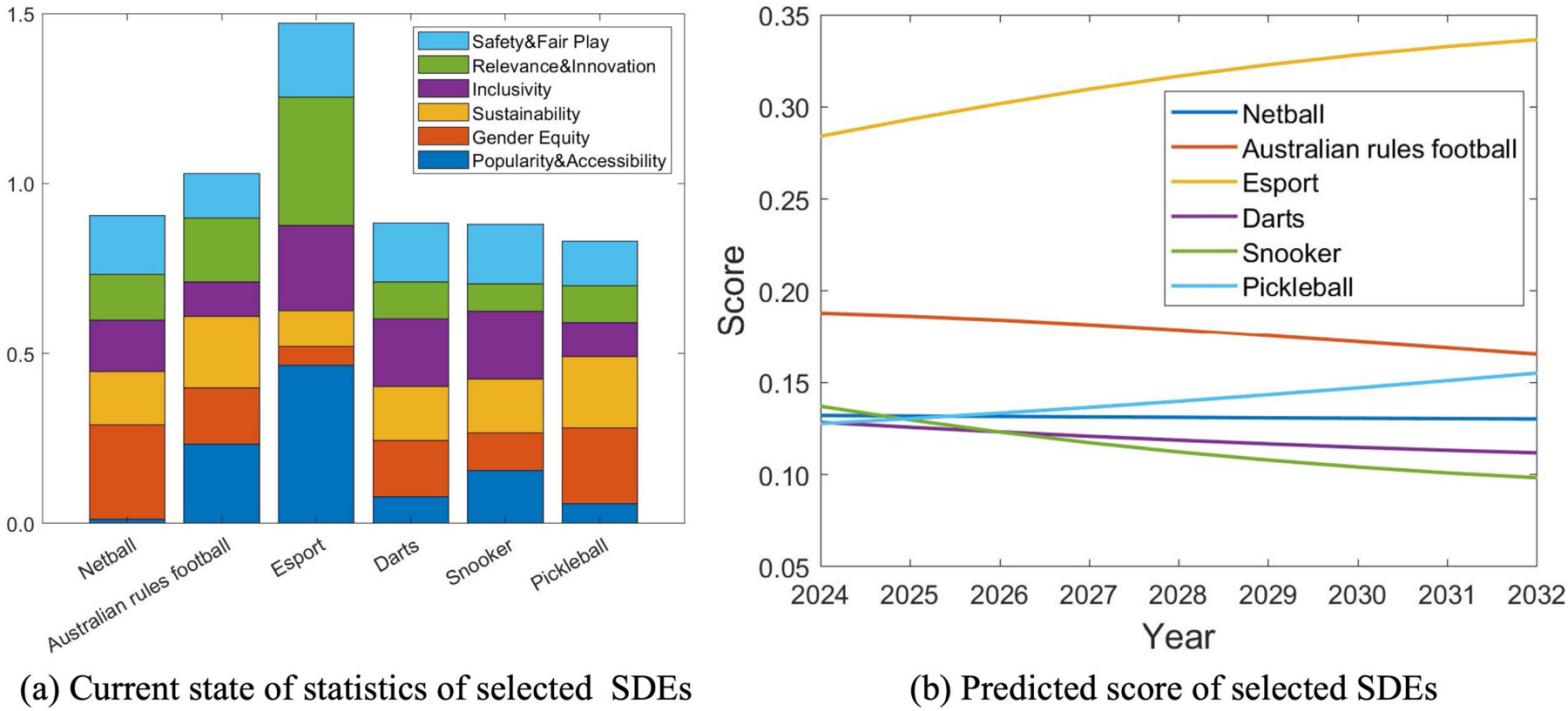
Current state and future estimates of 6 candidates for the 2032 Brisbane Olympic Games. **(a)** Current state of statistics of selected SDEs and **(b)** Predicted score of selected SDEs.

Following our scoring system, the current state of statistics of selected sports is shown in Figure 9a. It can be seen that Esports has the largest combined score due to its high popularity, inclusivity and youth appeal. Australian rules foot ball has wide audiences and is also competitive in terms of sustainability. Besides, although pickleball is a relatively young sport, it has a high level of gender equity and sustainability. To investigate which sports are more suitable for the 2032 Olympics, we study their change over time. As well, the most important and also most volatile factor is popularity. According to (28, 29) and also data fetched via social media (18), the estimated average growth of popularity of the selected SDEs in the past three years is listed in Table 5. Based on this observation and prior knowledge, we can apply our scoring system and calculate the estimated scores of different years by taking consideration of such variations. Figure 9 shows the change of the scores over time. It can be seen that the top 3 candidates for the 2032 Brisbane Olympic Games are Esports, Australian rules football and Pickleball. Furthermore, for the 2036 Olympic Games and beyond, we believe that tug of war, speed chess and bowling should be included for their global popularity, gender equity, safety and also appeal to people across all age groups.

**Table 5:** Average growth rate of 6 selected sports.

SDE	Netball	Australian rules football	Esports	Darts	Snooker	Pickleball
Growth rate	1.15	1.2	1.3	1.1	1.05	1.4

### 4.4 Sensitivity analysis: question 5

**Figure 10:**
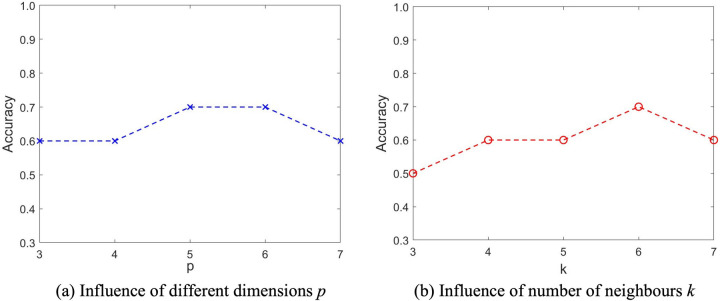
Sensitivity and parameter analysis of feature size p and number of nearest neighbours k. Influence of **(a)** different dimensions p and **(b)** number of neighbours *k*.

In our model and experiments, the low-dimensional embedding feature size p and the number of nearest neighbours k play a crucial role, thus in this section we perform sensitivity analysis to investigate their impacts on the predicted results in terms of classification accuracy. Specifically, we randomly select 80% of data from D as our training set $D_{\mathrm{train}}$, and the rest 20% are used for testing. Figure 10 compares the classification accuracy with different p and k. Interestingly, it can be seen that increasing the dimension size p does not always bring about benefits as more eigenvectors may capture not only meaningful variance but also noise. Similarly, choosing a larger number of neighbours k increases the risk of misclassification, in that it may be difficult to find sufficient neighbours that share similar features. In practice, we can select p and k from 5 to 7 for better predictions.

## 5 Discussion

### 5.1 Evaluation of the model’s performance

In this paper, we have proposed a comprehensive scoring and labelling system for evaluating SDEs for the Olympics, taking into account a variety of criteria such as popularity, gender equity, sustainability, and safety. The system was further integrated into a PCA-based classification model, combining unsupervised learning for feature extraction with a supervised KNN classifier to provide a more robust and objective method for SDE selection. Our experimental results successfully highlighted the current status of a wide range of SDEs in the Olympic context, validating the model’s capability to categorize and prioritize sports based on IOC guidelines. Our analysis identified Esports, Australian rules football and pickleball as top contenders. Our framework provides valuable insights for future Olympic event evaluations and can inform decisions for the 2032 Brisbane Olympics. Finally, these findings carry important policy implications that should be considered from multiple perspectives, including those of the IOC, international sport federations, and potential host cities, to ensure balanced, sustainable, and strategically aligned event portfolios.

### 5.2 Implications for Olympic programme planning

Beyond the technical evaluation of SDEs, the planning of the Olympic programme must be firmly grounded in the broader mission of the Olympic Movement. As articulated in the Olympic Charter and reinforced by recent scholarship, the Olympic Games serve not only as a stage for elite competition but also as a global platform for promoting fundamental values such as excellence, friendship, and respect (14). These core principles should fundamentally guide decisions on sport inclusion, ensuring that new disciplines enhance public engagement, foster participation, and contribute to the Games’ lasting social and cultural impact.

Consistent with this mission, the Olympic Movement has long promoted global sport through initiatives like the Olympic Values Education Programme (OVEP), which cultivates moral awareness, cultural understanding, and essential life skills (30). In recent years, the Olympic Games have also faced heightened scrutiny regarding issues such as safety, pandemic management, environmental sustainability, and gender equality (14).

The revitalization of OVEP should be guided by the principles of Education for Sustainable Development (ESD), which equip individuals with the knowledge, values, and competencies needed to address global challenges through a sustainability lens (14, 15, 31). Embedding ESD objectives—such as climate literacy, equitable resource access, and environmental responsibility—into OVEP’s curriculum and pedagogy would allow the programme to foster ethical decision-making, intercultural understanding, and long-term sustainability competencies (15, 31). These principles can also be extended beyond education into practice, particularly in the area of sustainable urban planning. For example, both Beijing2008, London 2012 and Sochi 2014 integrated Olympic investments into long-term infrastructure development, transforming sporting venues and public spaces into lasting community assets (31). By aligning OVEP with such broader sustainability initiatives, the IOC can ensure that Olympic education not only transmits values but also supports systemic change across social, environmental, and urban domains.

Further reinforcing this commitment, the IOC’s endorsement of initiatives like the Hamburg Declaration, in collaboration with the World Health Organization, highlights the role of sport in promoting public health and sustainability (13). This underscores the need to prioritize sports that encourage daily physical activity and community sport (32). As (14) note, maximizing the IOC’ prestige and momentum entails selecting disciplines with low barriers to entry (e.g., swimming, cycling, running) and requiring host cities to invest in accessible community sports infrastructure.

While the current model emphasizes quantifiable factors such as media visibility, gender equity, and global reach, future iterations should incorporate indicators related to sustainability, education, and health. By combining rigorous data-driven analysis with ethical and philosophical perspectives, the IOC can more effectively align sport selection with its evolving responsibilities in the 21st century. This holistic approach will help foster a more inclusive, sustainable, and forward-looking Olympic legacy.

### 5.3 Limitations and future directions

While the proposed method demonstrates effectiveness, there are areas where improvements can be made. For instance, during the scoring and labeling phase, incorporating additional factors such as athleticism, game duration, and historical significance of the sport could lead to a more comprehensive evaluation. Furthermore, the application of more advanced machine learning techniques, such as Support Vector Machines (SVM) (33) and Deep Learning (DL) (34), could enhance the accuracy and robustness of the classification model, allowing for better predictions and more nuanced decision-making.

Future work should focus on extending the model by integrating real-time data to capture shifts in public engagement and sport trends, as well as exploring other advanced algorithms to refine the classification process. Additionally, expanding the datasets to include emerging sports will help improve the model’s adaptability. Emerging sports are defined here as disciplines that have recently gained international visibility, institutional support, or rapid growth in participation. These sports are not yet part of the official Olympic program. Furthermore, incorporating feedback from stakeholders will also contribute to ensuring the model remains adaptable to the dynamic landscape of the Olympic Games. However, due to limited availability of comprehensive stakeholder data and real-time IOC decisions, our current study does not include external validation based on such inputs. We acknowledge this as a limitation and suggest that future work could strengthen the model’s reliability by integrating actual feedback from the IOC, international sport federations, and potential host cities when such data becomes accessible.

In parallel, while this study focuses on the Summer Olympic Games, the proposed framework could be extended to the Winter Games by adapting the evaluation criteria to reflect the distinct characteristics of winter sports, such as climatic dependence, snow- and ice-specific infrastructure, and limited geographic accessibility. Accounting for these factors represents a promising direction for extending and validating the model in broader Olympic contexts.

Finally, we note that some indicators, such as social media metrics or broadcast coverage, emphasize visibility over grassroots participation. While aligned with the IOC’s focus on youth engagement and media reach, they may overlook factors like historical significance or adaptability (e.g., the evolution of modern pentathlon). These qualitative aspects were excluded due to difficulties in quantification, but future studies could incorporate them through expert input or case-based methods.

## Data Availability

The original contributions presented in the study are included in the article/Supplementary Material, further inquiries can be directed to the corresponding author/s.
