# Supplementary material for: Quantifying future Olympic sport selection: a data-driven framework for SDE evaluation and selection
Source: Front Sports Act Living. 2025 Jul 29;7:1596196. doi: 10.3389/fspor.2025.1596196 (PMC12339490; doi:10.3389/fspor.2025.1596196)
Supplement: Supplementary file 1 [file Datasheet1.pdf]

Supplementary Material

1 SUPPLEMENTARY TABLES AND FIGURES

1.1 Tables

Table S1. Evaluations of recently added or removed SDEs.

SDE	Score	Ranking	Expected label (category)	Predicted label (category)	Current status
Breakdancing	0.0214	25	0 (Moderate)	-1 (Low)	Removed (2028)
Cricket	0.0319	6	1 (High)	1 (High)	Added (2028)
Flag football	0.0179	39	-1 (Low)	-1 (Low)	Added (2028)
Basketball (3x3)	0.0259	11	1 (High)	1 (High)	Added (2020)

Table S2. Evaluations of SDEs continuously been in the Olympics since the 1988 games or earlier.

SDE	Score	Ranking	Expected label (category)	Predicted label (category)	Current status
Tennis	0.0334	3	1 (High)	1 (High)	Added (1988)
Fencing	0.0207	19	0 (Moderate)	0 (Moderate)	Added (1896)
Judo	0.0188	27	0 (Moderate)	-1 (Low)	Added (1972)
Weightlifting	0.0187	28	-1 (Low)	-1 (Low)	Added (1920)

1.2 Figures

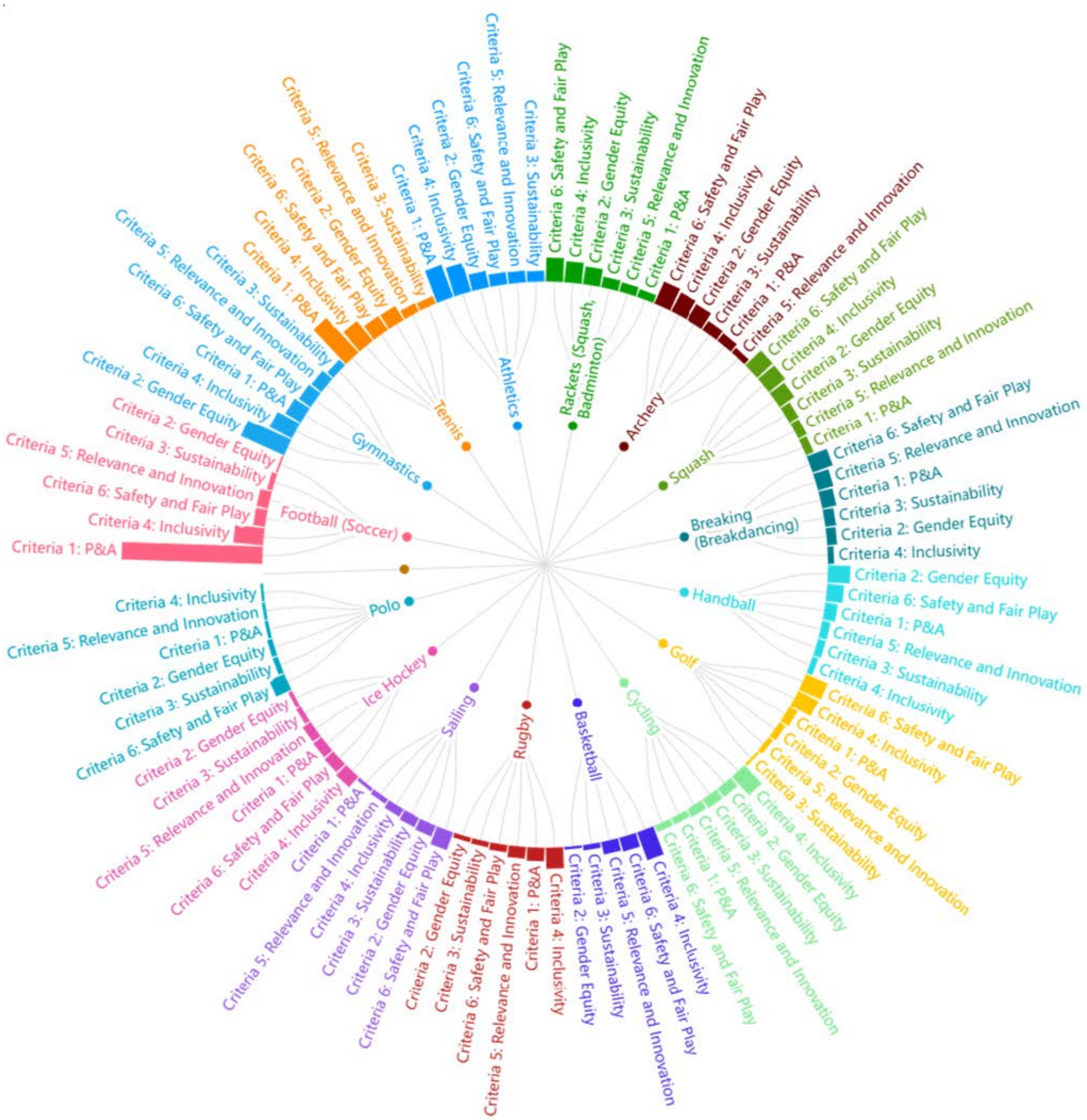

Figure S1. Representative SDEs and their corresponding values of different criteria.

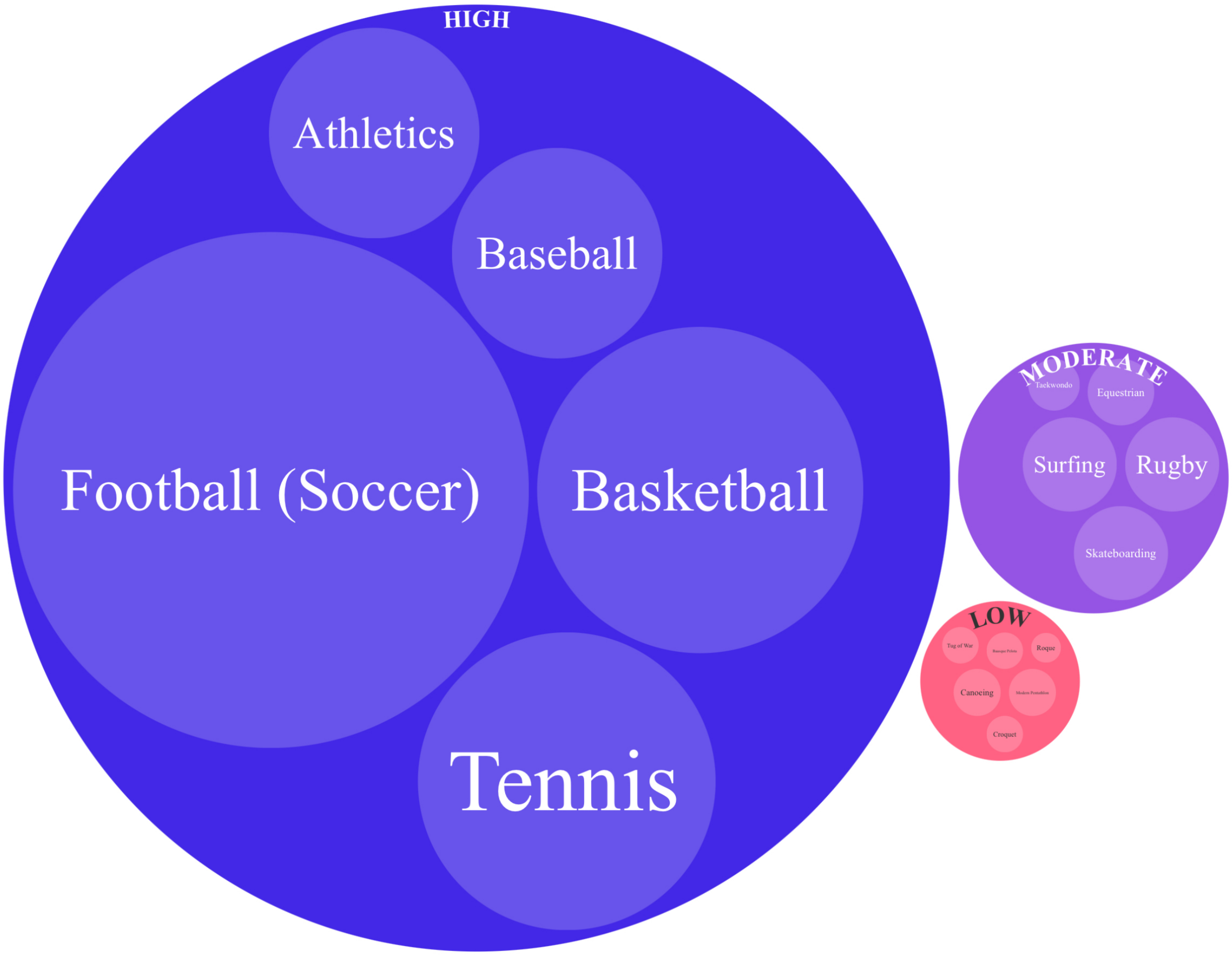

Figure S2. Popularity of different SDEs.

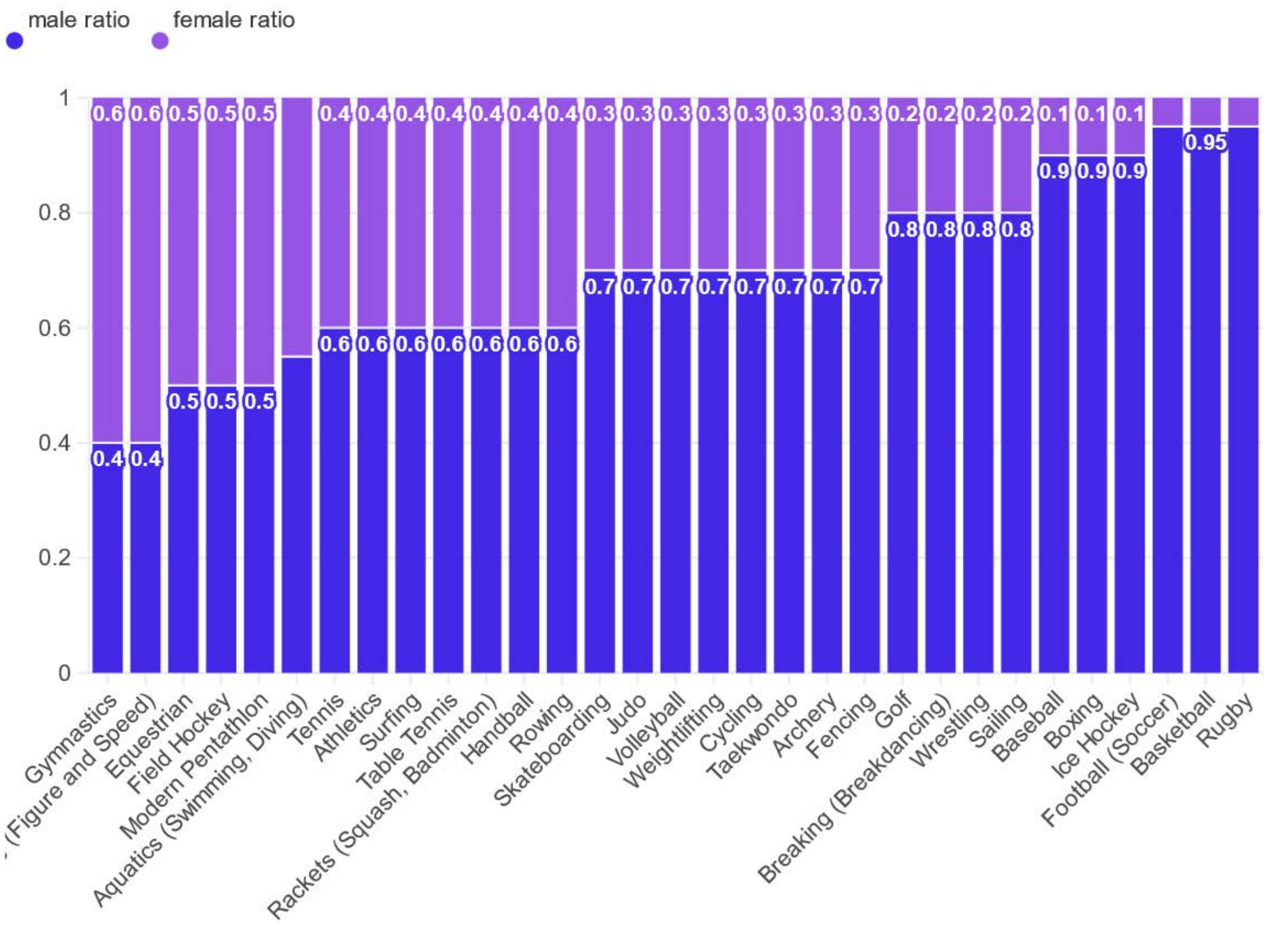

Figure S3. Illustration of gender equity measured by the income ratio and participant rate.

```
function A_normalized = normalization(A)

[num_sports, num_features] = size(A);
A_normalized = zeros(num_sports, num_features);

for i = 1:num_features
    A_feature_i = A(:,i);
    sum_feature_i = sum(A_feature_i);
    A_normalized(:,i) = A_feature_i/sum_feature_i;
end

end
```

Figure S4. Implementation of data rescaling and normalization.

```
load("data_sum_normalized_combined_by_sum.mat");
load weight_AHP

data = A_sum_normalized_combined;
weight = weight;

weighted_score = data*weight';
[~,sort_idx] = sort(weighted_score,'descend');
num_sports = length(weighted_score);
ranking_all = zeros(num_sports,1);

for i = 1:num_sports
    rank_idx_i = sort_idx(i);
    ranking_all(rank_idx_i) = i;
end
```

Figure S5. Implementation of SDE scoring and labeling system.

```
function [Uk] = My_PCA(X, k)
% The input X is an N by D matrix
% where N is the number of samples, and D is the feature size
% k represents the number of eigenvectors or PCs you want to select

% Step 1: Centralization (obtain mean value by row)
X_mean = mean(X);
% Step 2: Calculate covariance matrix Z
[N,d] = size(X);
Z = (1/(N-1)) * (X - X_mean)' * (X-X_mean);
% Step 3: Perform eigendecomposition to obtain eigenvalues/eigenvectors(PCs)
[Uk,~] = eigs(Z, k);

end
```

Figure S6. Implementation of PCA for feature extraction and dimensionality reduction.

```
function [Predicted_labels] = KNN_classfier(Train_data, Test_data, Train_labels, K)
% KNN_CLASSFIER

num_train_data = size(Train_data,1);
num_test_data = size(Test_data,1);
Predicted_labels = zeros(num_test_data,1); % initialize

for i = 1:num_test_data % 80

    % Fetch the i-th test data
    Test_data_i = Test_data(i,:);
    % Compare it with all training data
    distance = zeros(num_train_data, 1);
    for j = 1:num_train_data
        Train_data_j = Train_data(j,:);
        % Calculate Euclidean distance between test data and every training data
        distance(j) = norm(Test_data_i(:) - Train_data_j(:));
    end

    % Sort the distance according to ascending order (from cloest to the furthest)
    [~, sort_idx] = sort(distance, 'ascend');
    % Now we only need K nearest neighbours
    First_K_idx = sort_idx(1:K);
    Train_labels_first_K_neighbours = Train_labels(First_K_idx);
    % Then we vote for the best one (Just find the mode!!!).
    mode_label = mode(Train_labels_first_K_neighbours);
    % Then the mode should be our prediction
    Predicted_labels(i) = mode_label;
```

Figure S7. Implementation of KNN classifier.
